# Operational challenges of industrial wastewater treatment plants in a multi-river province of China: implications for problem identification and management

**DOI:** 10.3389/fchem.2026.1955505

**Published:** 2026-09-23

**Authors:** Zhilong Dong, Jing Zhou, Nan Li, Yi Huang, Fengli Tang, Long Sun, Wei Tao, Bowen Dong, Chengcheng Wen

**Affiliations:** 1 Gansu Appraisal Center for Ecology Environment and Engineering, Lanzhou, China; 2 Gansu Academy of Eco-environmental Sciences, Lanzhou, China; 3 Key Laboratory of Industrial Waste Recycling and Regulation, Gansu Academy of Eco-environmental Sciences, Lanzhou, China; 4 Key Laboratory of Northwest Water Resource, Environment and Ecology, MOE, Xi’an University of Architecture and Technology, Xi’an, China; 5 Shaanxi Provincial Field Scientific Observation and Research Station of Water Quality in Qinling Mountains, Xi’an University of Architecture and Technology, Xi’an, China

**Keywords:** industrial WWTPs, multi-river province, operational countermeasures, operational management, operational problems

## Abstract

**Introduction:**

Industrial wastewater treatment plants are the primary sources of pollution in receiving water bodies, and their operational status directly affects the river water quality security, especially in provinces with multiple rivers. However, most reports focus on individual case studies and fail to conduct systematic research across basin and regional scales to support water environment risk assessment and control for decision-making and administrators.

**Methods:**

Given this, this study aimed to sort out the operational problems of industrial WWTPs and corresponding countermeasures at both basin and provincial scales, based on a 3-year investigation conducted in Gansu Province.

**Results:**

A comprehensive profile of operational problems, involving three common and 15 specific aspects, was developed at both basin and provincial scales. At the provincial scale, operational problems of WWTPs mainly included high dependence treatment rate (50.0%), low-load operation in terms of influent quantity (47.4%) and quality (e.g., chemical oxygen demand: 41.7%), a high proportion of WWTPs with a compliance rate below 90% (34.4%), high unit sludge yield (2.02 t/10^4^ m^3^), and high operational costs (3.91 CNY/m^3^). Major operational problems of WWTPs varied across the three basins. For example, design- and influence-related problems were more prominent in the Yellow River basin, whereas influentrelated and actual-operational performance problems were more prevalent in the Northwest River basin. Low loads of influent quantity and quality were the primary factors influencing the operational performance of WWTPs, followed by the operational level and design. These results suggest that for enhancing the operational efficiency of industrial WWTPs, three important steps are required to be implemented: (i) optimizing operational performance, (ii) increasing influent loads in terms of quantity and quality, and (iii) addressing design deficiencies.

**Discussion:**

Collectively, these measures can facilitate a shift from an effluent compliance-oriented approach to comprehensive process-based optimization and management. This study also provides a useful framework for investigating industrial WWTPs and identifying and addressing operational challenges.

## 1 Introduction

With the rapid socio-economic development of China, the conflict between water resource conservation and economic growth has become increasingly evident, particularly in the country’s two major economic corridors associated with the Yellow River (YR) and the Yangtze River (YTR) (Pan et al., 2020). Therefore, a series of national policy documents have been implemented to prevent basin water pollution, particularly since 2012 (Mao et al., 2019). As a result, the proportion of good-quality water has increased by 15.7% in the YR basin and 2.6% in the YTR basin (Wei et al., 2022). Consequently, the focus of river water environment management has gradually shifted from conventional water pollution control to improving water quality in heavily polluted tributaries and mitigating river water environment risks associated with anthropogenic sources such as industrial wastewater discharge (Long et al., 2018). Further, several targeted documents have been implemented to enhance the upgrading and renovation of industrial wastewater treatment plants (WWTPs) in environmentally sensitive areas of multi-river provinces to reduce water environment risks and support sustainable basin development (Wei et al., 2022; Zhang et al., 2026). In this context, large-scale studies on the operation of industrial WWTPs are needed to support the development of integrated river pollution risk prevention and management system at the provincial, basin, and national levels.

In response to the national major policy deployments of river pollution risk prevention systems, provincial governments in China have issued a number of policies to promote governance and study of the operational shortcomings of industrial WWTPs. Indeed, some study achievement of WWTP operation in China was obtained, with the increasing rate of publishment by six papers per year (Supplementary Figure S1). However, several gaps remain in the existing literature. First, from the perspective of research scales, most studies focus on individual case studies (Wei et al., 2022; Zhang et al., 2026); however, large-scale studies including provincial-, basin-, and national-scale remain relatively limited. Second, from the perspective of research focus, most studies mainly examine specific aspects of WWTPs operation, such as chemical and energy consumption, operational cost, wastewater and sludge recycling, and treatment efficiency enhancements (Awad et al., 2019; Qi et al., 2020; Qu et al., 2025); however, systematic review studies remain relatively limited. Third, most previous studies can effectively identify the operational challenges of WWTPs (Lu et al., 2019; Zhang et al., 2016); however, they fail to provide targeted recommendations for addressing these challenges. Currently, only a few provinces and river basins have conducted systematic investigations of the operational challenges of industrial WWTPs, limiting the identification, assessment and prevention of river pollution risk from industrial WWTPs at the national scale.

In addition, several practical and methodological challenges hinder the research on the operation of industrial WWTPs. For example, (a) for such field studies, only the broad-scope and long-period survey can generate valuable conclusions, but obtaining long-term and large-area data is rather difficult; (b) unlike conventional field studies, capturing high-frequency data set is likewise troublesome in consideration of the revelation of local operational effectiveness of WWTPs; (c) it is difficult to summarize a reliable systematic study and propose valuable countermeasures through a few cases due to the complexity of the research subjects, especially for industrial WWTPs with complex wastewater quality and treatment processes (Long et al., 2018; Zhang et al., 2021).

Furthermore, owing to the different representative problems among provinces and basins, other successful experiences cannot be simply applied to guide local practices (Wei et al., 2022). Therefore, considering the uniqueness of the research subjects and research limitations triggered by the above-mentioned factors, one-area-one-strategy studies on industrial WWTPs’ operation based on high-frequency, long-term, and large-area surveys should be implemented, especially in the upstream provinces of river basins.

Gansu Province, known as the upstream province of the China’s main rivers, such as the YR, YTR, and Northwest River (NWR) basins (Sun et al., 2023), is considered as a research subject to improve the local operational efficiency of industrial WWTPs and reduce the water pollution risk of rivers, primarily due to its unique geographical position and the lack of related research. Therefore, we conducted 3-year field investigation of 70 industrial WWTPs distributed in the YR, YTR, and NWR basins of Gansu Province. The study aimed to identify both common and basin-specific operational problems of WWTPs and to propose feasible improvement strategies. The objectives of our study were to (1) examine potential water pollution problems based on the investigation of the layout, construction, and design of industrial WWTPs; (2) identify the actual operational problems of up-to-standard discharge and efficiency improvement based on the investigation of inlet and outlet water quality characteristics, pollution control performance, unit sludge yield, and operational cost; and (3) propose one-to-one operational countermeasures based on the above potential and actual problems to improve the operational efficiency of local WWTPs and support the development of one-place-one-strategy research system.

## 2 Materials and methods

### 2.1 Study areas

The investigation was conducted at industrial parks in a multi-river province of China, which included 53 industrial parks (7 national-level and 46 provincial-level parks) and 70 WWTPs (35 industrial WWTPs, 33 municipal WWTPs, and 2 corporate WWTPs) distributed across different river basins of Gansu Province. This province is known as a typical high-altitude and low-temperature area across the entire country, with the lower average air temperature (reduction of about 1.3 °C) and its longer duration period (increase of about 1 month) in China, which can induce the operational challenges involving WWTP insulation and related increase in energy consumption and operational cost (detailed discussion provided in Section 4.1).

Among the studied WWTPs, 42 WWTPs, 23 WWTPs, and 5 WWTPs were distributed in the YR, NWR, and YTR basins, respectively, and the median distance between WWTPs and rivers was approximately 0.7 km. These WWTPs are characterized by a high-density distribution within the river basins and close proximity to rivers (Supplementary Figures S2b,c), resulting in persistent substantial discharge to receiving water bodies and increasing the potential risk to the river water environment.

### 2.2 Study methods

The systematic study approach is illustrated in Figure 1. First, systematic data was collected to avoid superficial research on the operation of industrial WWTPs by combining a questionnaire survey with field investigations, and an all-sided dataset was used to investigate the operational problems and associated risks to the river water environment from plant construction to actual operation. Second, the study area was divided into two levels (i.e., total and basin levels) to precisely identify the common and specific problems of the river water environment caused by the operation of WWTPs, which could be helpful for the identification and risk management of river water pollution. Third, the operational problems were categorized into two types (i.e., main and detailed problems) to identify the true causes, and their connections were further established to facilitate the formulation of targeted countermeasures to promote water environment risk management. Moreover, an overview of the operational problems and countermeasures of WWTPs was developed at both the total and basin scales to support river water risk management (Figure 2).

**FIGURE 1:**
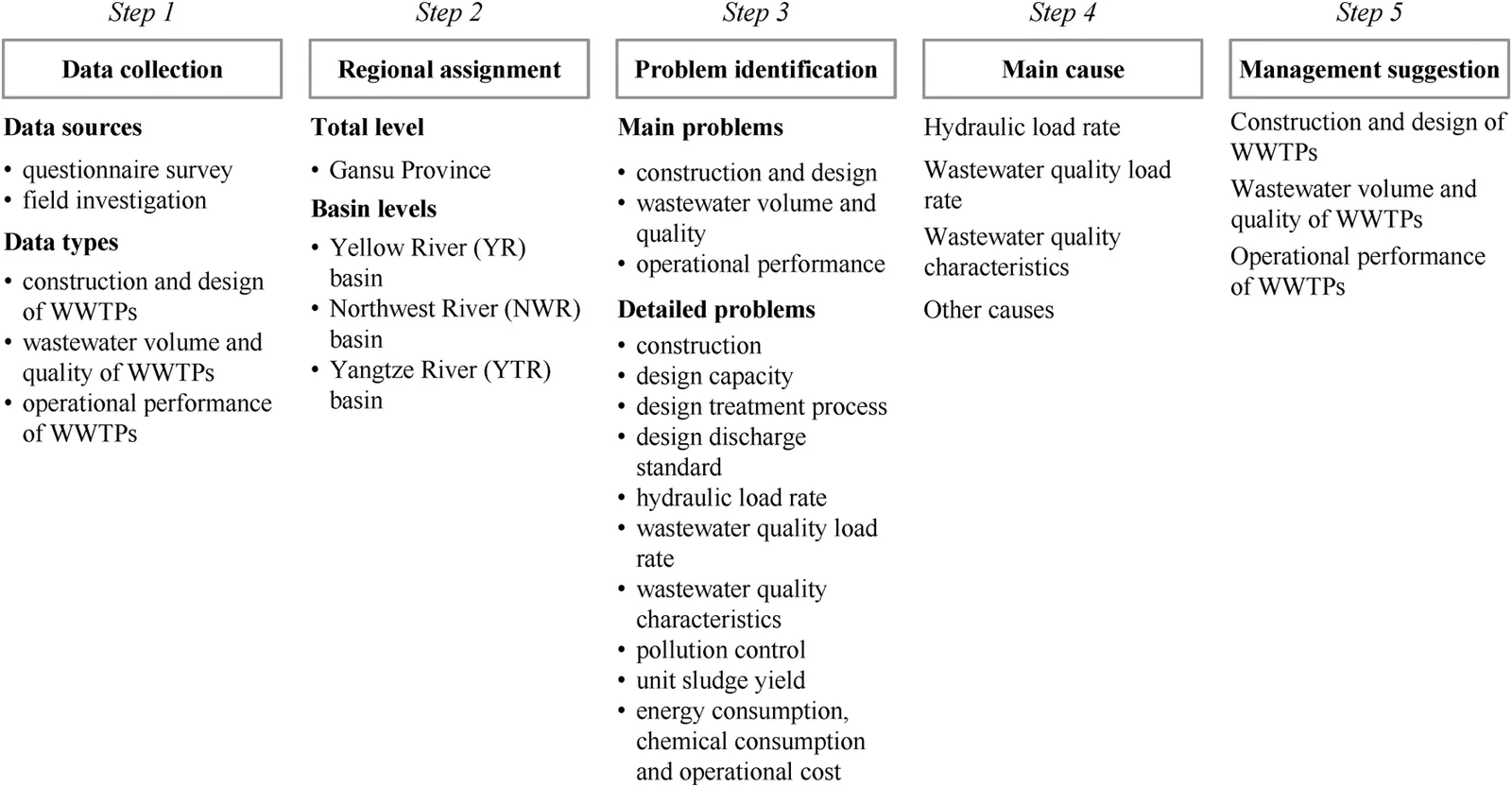
Study approach of operational challenges of WWTPs in a multi-river province of China.

**FIGURE 2:**
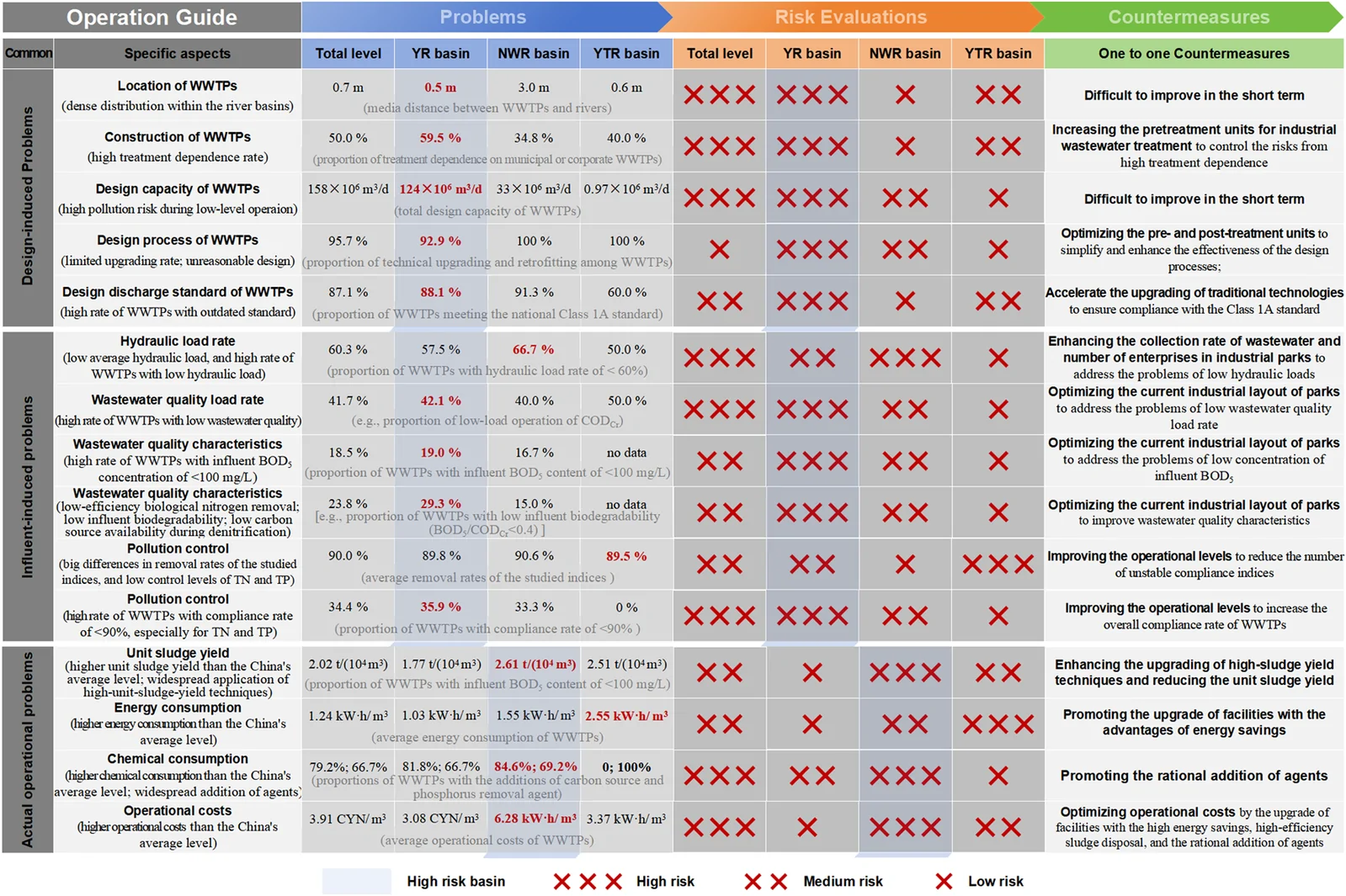
An overview of operational problems and countermeasures of industrial WWTPs at the total and basin levels in a multi-river province of China.

This study was conducted monthly from 2022 to 2024 using field investigations and questionnaire surveys, and related data analyses were based on the annual operational status of WWTPs.

### 2.3 Current status of the WWTPs construction and design

#### 2.3.1 Construction of the WWTPs

Since 1988, the construction of industrial parks has undergone three stages: initial, booming, and stable (Supplementary Figure S2a). Currently, there are 53 provincial-level and above industrial parks in Gansu Province, of which 86.8% are comprehensive types and 13.2% are chemical types, all of which have been collected and treated through 70 WWTPs. However, 50% of the wastewater treatment relies on municipal or corporate WWTPs, and 28.6% relies on chemical industrial parks. Furthermore, the proportions of wastewater treatment reliance in the YR, NWR, and YTR basins are 59.5%, 34.8%, and 40.0%, respectively. In conclusion, industrial WWTPs exhibited common challenges at both overall and basin scales, including inadequate infrastructure development and a high dependence on municipal or corporate WWTPs. This issue was particularly prominent in chemical industrial parks.

#### 2.3.2 Design capacity of the WWTPs

The designed capacity of WWTPs in Gansu Province ranged from 200 m^3^/d to 3 × 10^5^ m^3^/d, with a total of 1.58 × 10^8^ m^3^/d and an average of 2.26 × 10^4^ m^3^/d. The total design capacity of the WWTPs in the YR, NWR, and YTR basins accounted for 78.4%, 21.0%, and 0.6% of the provincial total, respectively, with average levels being 1.3, 0.4, and 0.1 times that of the provincial average (Supplementary Figure S3a). Furthermore, their design scale mainly focused on micro- (40%), small- (21.4%), and medium-scale (35.7%) WWTPs, with a relatively low proportion of large WWTPs (2.9%). The YR basin was dominated by micro- (26.2%), small- (26.2%), and medium-scale (42.9%) plants; the NWR basin was dominated by micro- (56.5%) and medium-scale (30.4%) plants; and the YTR basin was dominated by micro- (80%) and small-scale (20%) WWTPs (Supplementary Figure S3b). Differences in the treatment scale of WWTPs in the three basins were observed, but the total volumes in the YR and NWR basins were higher than those in the YTR basin, implying that the higher risks to water environmental safety can be induced by the poor efficiency of these WWTPs.

#### 2.3.3 Treatment process design of the WWTPs

Most WWTPs in Gansu Province (95.7%) have been upgraded primarily through the addition of advanced treatment technologies. In addition, all WWTPs in the NWR and YTR basins have completed technical upgrading and retrofitting; however, 7.1% of the WWTPs in the YR basin still use traditional treatment processes (Supplementary Figures S4a,c,e).

Two WWTP upgrading modes were observed. One treatment mode incorporated enhanced technologies only during the advanced treatment, accounting for 95.7% of the total. However, among them, the proportion of high-energy-consumption, high-operation-cost, and high-complexity processes (e.g., Membrane Bio-Reactor, Sedimentation Tank+Advanced Oxidation Process+Filters) was high (41.4%), especially in the NWR basin (Supplementary Figure S4d). Another treatment mode incorporated enhanced technologies during both pretreatment and advanced treatment stages, accounting for 42.9% of the total (Supplementary Figures S4b,d). The enhanced technologies used during pretreatment included two methods. The first method involved the addition of membrane grille or high-efficiency settling tanks to remove the total amount of particulate matter; however, this may reduce the availability of carbon sources for the secondary treatment units. The second method involved the addition of high-efficiency anaerobic tanks or their combined processes to remove refractory particulate matter. This was an appropriate approach for improving wastewater biodegradability. However, such process arrangements neglect the fact that WWTPs in Gansu Province mainly treat wastewater from comprehensive parks with low pollution loads, resulting in overdesign. Instead, a low proportion of WWTPs in chemical industrial parks incorporated high-efficiency anaerobic tanks during pretreatment (14.3%), which led to insufficient design (Supplementary Figure S4b).

Overall, the technical upgrading of WWTPs in Gansu Province has achieved remarkable results; however, some issues have also been identified. For instance, during the design stage, the most complex pretreatment and advanced treatment processes are adopted to ensure effluent quality, resulting in overdesign and increased operational energy consumption. Additionally, the enhanced technologies incorporated during pretreatment, especially for chemical industrial parks, are either lacking or insufficiently targeted, indicating shortcomings in the design stage. During the actual operation stage, owing to the insufficient number of enterprises entering the parks, low-loading-rate phenomena of both wastewater volume and quality have generally emerged, which results in insufficient carbon sources in the influent and increased chemical consumption cost.

#### 2.3.4 Design discharge standard of the WWTPs

Among the discharge standards for WWTPs in Gansu Province, the national Class 1A standard, Class 1B standard, and other standards accounted for 87.2%, 5.7%, and 7.1%, respectively (Supplementary Figure S5a). Therein, the upgrading of WWTPs in the NWR basin achieved the best results (91.3% of WWTPs met the Class 1A standard), followed by those in the YR basin (88.1% of WWTPs met the Class 1A standard) and in the YTR basin (60% of WWTPs met the Class 1A standard) (Supplementary Figures S5b–d).

Further considering the design processes and discharge directions of WWTPs (Supplementary Figures S4, S5), the WWTPs operating under lower environmental standards can be divided into three categories: (1) the first category, namely, outdated discharge standard, advanced technology, and no direct discharge of pollutants to rivers, is found in 8.7% of WWTPs in the NWR basin and 40% of WWTPs in the YTR basin; (2) the second category, namely, outdated discharge standard, advanced technology, and direct discharge of pollutants to rivers, is found in 2.4% of WWTPs in the YR basin; and (3) the third category, namely, outdated discharge standard, outdated technology, and direct discharge of pollutants to rivers, is found in 9.5% of WWTPs in the YR basin. Clearly, the water environment risks from WWTPs in the NWR and YTR basins are lower than those in the YR basin, suggesting a need for process upgrades and improved discharge standards in WWTPs located within the YR basin.

## 3 Results

### 3.1 The operational performance of the WWTPs

#### 3.1.1 Pollution control

The average influent concentrations of suspended solids (SS), 5-day biochemical oxygen demand (BOD_5_), chemical oxygen demand (COD_Cr_), total nitrogen (TN), ammonia nitrogen (NH_3_-N), and total phosphorus (TP) in WWTPs in Gansu Province were 295.7, 174.2, 411.8, 60.0, 40.9, and 5.8 mg/L, respectively. In addition, the main pollution indices were different in the WWTPs of the three basins, such as SS, TN, and NH_3_-N of the WWTPs in the YR basin and BOD_5_, COD_Cr_, and TP of WWTPs in the NWR basin. More critically, the government has required WWTPs with influent BOD_5_ concentrations <100 mg/L to formulate improvement plans. Such WWTPs accounted for 18.5% of the province, 19.0% of the YR basin, and 16.7% of the NWR basin, respectively. The average effluent concentrations of SS, BOD_5_, COD_Cr_, TN, NH_3_-N, and TP in the Gansu WWTPs were 8.0, 24.7, 9.2, 1.4, 0.4, and 7.9 mg/L, respectively. This generally met the national Class 1A standard, and the WWTPs in the YTR basin had better performance than those in the other basins (Figure 3a). Nevertheless, except for COD_Cr_ and NH_3_-N, the effluent concentrations of the other indices still exceeded the Class 4 standard for surface water, suggesting a high-water environment risk of eutrophication and pollution.

**FIGURE 3:**
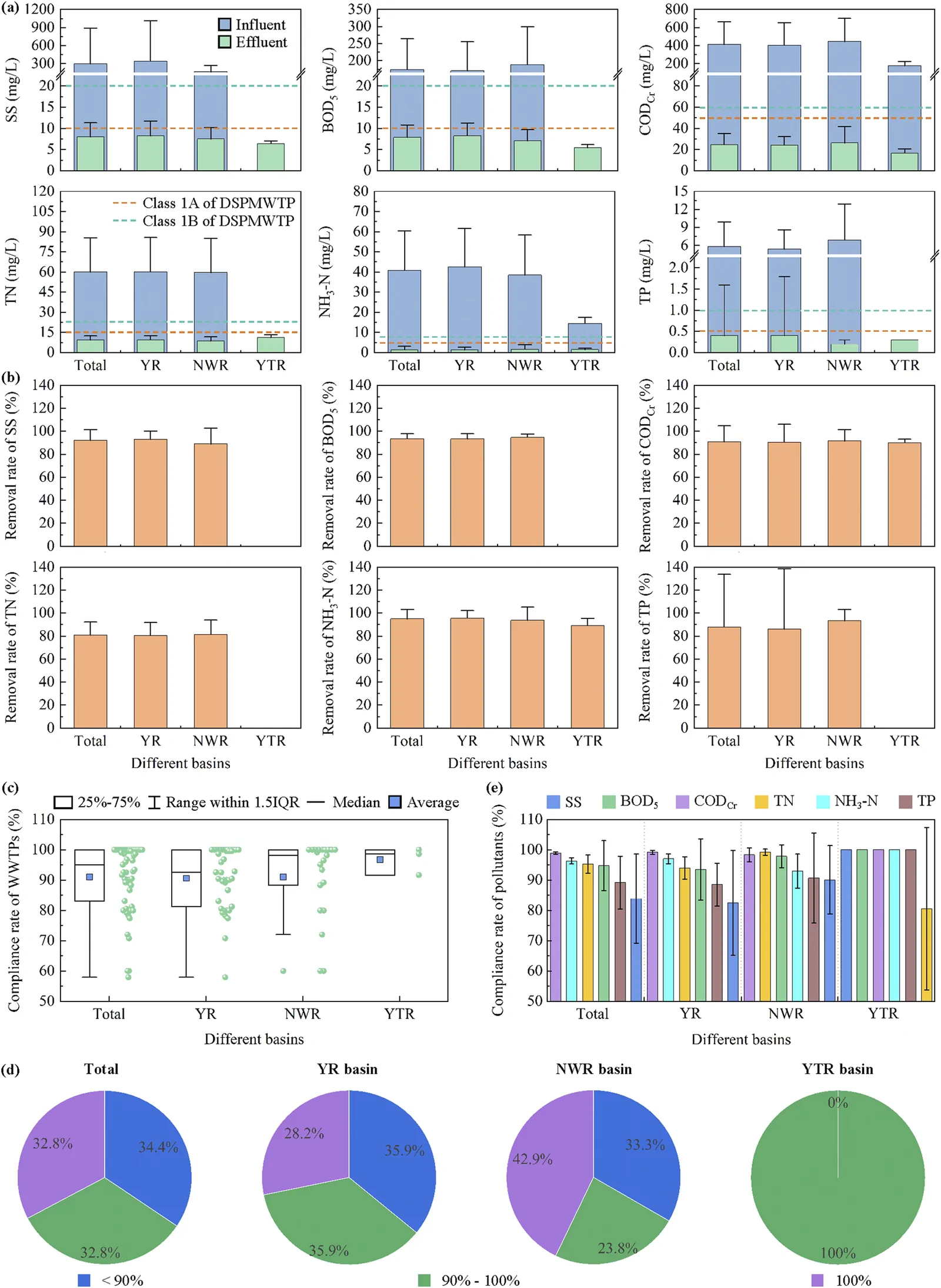
Treatment performance of WWTPs **(a)**, removal rate of pollutants **(b)**, compliance rate of WWTPs **(c)**, proportion of WWTPs with different compliance rate **(d)**, and compliance rate of pollutants **(e)** in a multi-river province of China.

Furthermore, the average removal rates of SS, BOD_5_, COD_Cr_, TN, NH_3_-N, and TP in the Gansu WWTPs were 92.0%, 93.5%, 90.8%, 80.7%, 95.0%, and 87.8%, respectively, and the control levels of TN and TP were lower than those of the other four indices. At the basin scale, WWTPs in the NWR basin showed the best pollutant control efficiency, followed by WWTPs in the YR and YTR basins (Figure 3b).

As a result, the compliance rates of Gansu WWTPs ranged from 57.9% to 100%, with an average of 91.0%, while the proportion of WWTPs with compliance rate of <90% was 34.4% (Figures 3c,d). At the basin scale, the WWTPs in the YTR basin had a higher compliance rate (96.8%) than those in the NWR (91.0%) and YR (90.6%) basins, and the proportions of WWTPs with compliance rate of <90% in the YR, NWR, and YTR basins were 35.9%, 23.8%, and 0%, respectively (Figures 3c,d). Meanwhile, unstable compliance indices (compliance rates of pollutants <95%), including SS, BOD_5_, TN, and TP, require particular attention throughout the province, and the main indicators differed among the three basins (Figure 3e).

#### 3.1.2 Unit sludge yield

The unit sludge yields of Gansu WWTPs ranged from 0 to 14.32 t/10^4^ m^3^, with an average value of 2.02 t/10^4^ m^3^, which were higher than the China’s average level (1.62 t/10^4^ m^3^). Therein, the average unit sludge yields of WWTPs in the NWR basin (2.61 t/10^4^ m^3^) were significantly higher than those in the YTR (2.51 t/10^4^ m^3^) and YR (1.77 t/10^4^ m^3^) basins, which were all higher than the China’s average level (Figure 4a). Furthermore, the unit sludge yields of the main technologies at the total and basin levels (e.g., OD and AAO) were also higher than China’s average (Figure 4b), especially for the WWTPs in the NWR basin. In general, the WWTPs in the different basins have some common problems, such as high unit sludge yields and poor operational efficiency, especially in the NWR basin, and the operational performance of technologies, such as OD and AAO, should be significantly improved.

**FIGURE 4:**
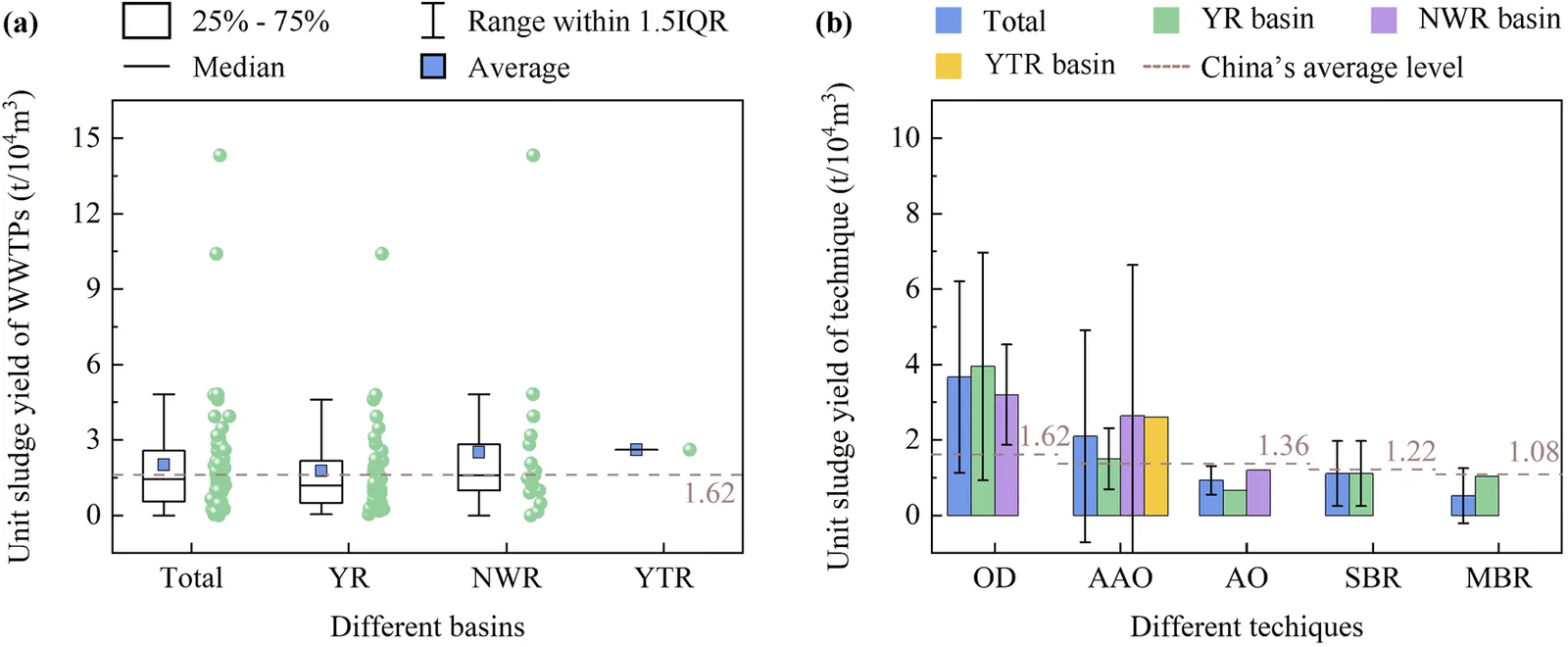
Unit sludge yield of total level **(a)** and different treatment techniques **(b)** of WWTPs in a multi-river province of China. Note: Anoxic-Oxic Process (AO); Anaerobic-Anoxic-Oxic Process (AAO); Membrane Bio-Reactor (MBR); Oxidation Ditch (OD); Sequencing Batch Reactor Activated Sludge Process (SBR).

#### 3.1.3 Energy consumption, chemical consumption, and operational costs

The energy consumption of Gansu WWTPs ranged from 0.03 to 8.49 kW h/m^3^, with an average value (1.24 kW h/m^3^) significantly higher than the China’s average (0.36 kW h/m^3^). Besides, the energy consumption of WWTPs in the YTR basin (2.55 kW h/m^3^) was significantly higher than those in the NWR (1.55 kW h/m^3^) and YR (1.03 kW h/m^3^) basins (Figure 5a).

**FIGURE 5:**
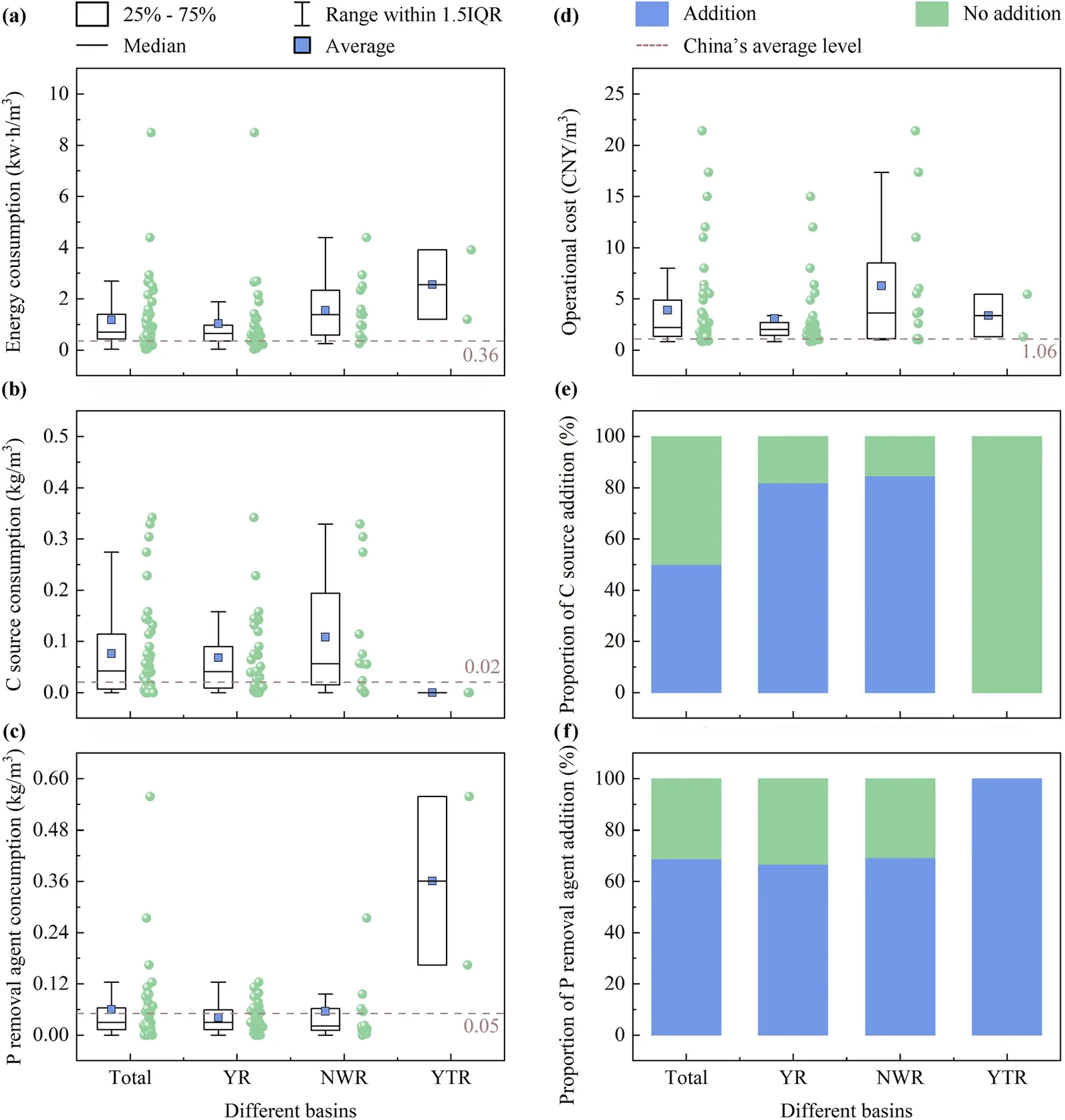
Energy consumption **(a)**, carbon source consumption **(b)**, phosphorus removal agent consumption **(c)**, operational cost **(d)**, and proportion of carbon source addition **(e)** and phosphorus removal agent addition **(f)** of WWTPs in a multi-river province of China.

The average unit consumption of carbon source and phosphorus removal agent was 0.08 and 0.06 kg/m^3^, respectively, which were markedly higher than the China’s average. Besides, the carbon source consumptions of WWTPs in the NWR basin (0.11 kg/m^3^) was significantly higher than those in the YR (0.07 kg/m^3^) and YTR (0 kW h/m^3^) basins, while the phosphorus removal agent consumption in the YTR basin (0.36 kg/m^3^) was significantly higher than those in the NWR (0.06 kg/m^3^) and YR (0.04 kW h/m^3^) basins (Figures 5b,c). The addition of carbon source and phosphorus removal agent was common in this province, with proportions of 79.2% and 66.7%, respectively (Figures 5e,f).

Moreover, the operational costs of Gansu WWTPs ranged from 0.82 to 21.40 CNY/m^3^, with an average value (3.91 CNY/m^3^) significantly higher than the China’s average (1.06 CNY/m^3^). At the basin scale, the operational costs of WWTPs in the NWR basin were the highest (6.28 CNY/m^3^), followed by those in the YTR (3.37 CNY/m^3^) and YR (3.08 CNY/m^3^) basins, and their average costs were 5.9, 3.2, and 2.9 times the China’s average, respectively (Figure 5d).

Overall, the average values of the above three indices for Gansu WWTPs were markedly higher than the China’s average, and the problems related to agent consumption were common at both the province and the basin scales. In addition, the main operational problems differed among the basins, with higher energy consumption observed in the YTR basin and the higher chemical consumption in the other basins.

### 3.2 Main factors influencing the WWTP operation

#### 3.2.1 Hydraulic load rate

Operational statuses can be divided into three categories: low-load operation (<60%), normal operation (loads within in the range of 60%–90%), and high-load operation (>90%, Ministry of Housing and Urban-Rural Development, 2015). Particularly, when WWTPs are operating at a low load level, corresponding low-load equipment operation and insufficient influent pollutant load (e.g., limited carbon source) will induce decrease in growth rate of activated sludge, relevant increase in hydraulic retention time, and reduction in pollutant removal efficiency (Zhang et al., 2016). Conversely, when WWTPs are operating at a high load level, accompanying overmuch wastewater quantity and quality can result in bulking of activated sludge, relevant reduction in hydraulic retention time, untreated wastewater overflows into river can excessive discharge of effluent and direct river water pollution (Qi et al., 2020).

The hydraulic loads of WWTPs ranged from 0% to 108.0%, with an average value of 47.4% (Figure 6a); the normal-operation WWTPs accounted for only 38.1%, while the low-load (<60%) and high-load (>90%) WWTPs accounted for 60.3% and 1.6%, respectively (Figure 6b). In addition, the average loads of the WWTPs in the YR, NWR, and YTR basins were 49.3%, 43.5%, and 61.7%, respectively (Figure 6a), whereas low-load operation WWTPs accounted for 57.5%, 66.7%, and 50%, respectively. Further, high-load operation WWTPs were observed only in the YR basin (Figure 6b).

**FIGURE 6:**
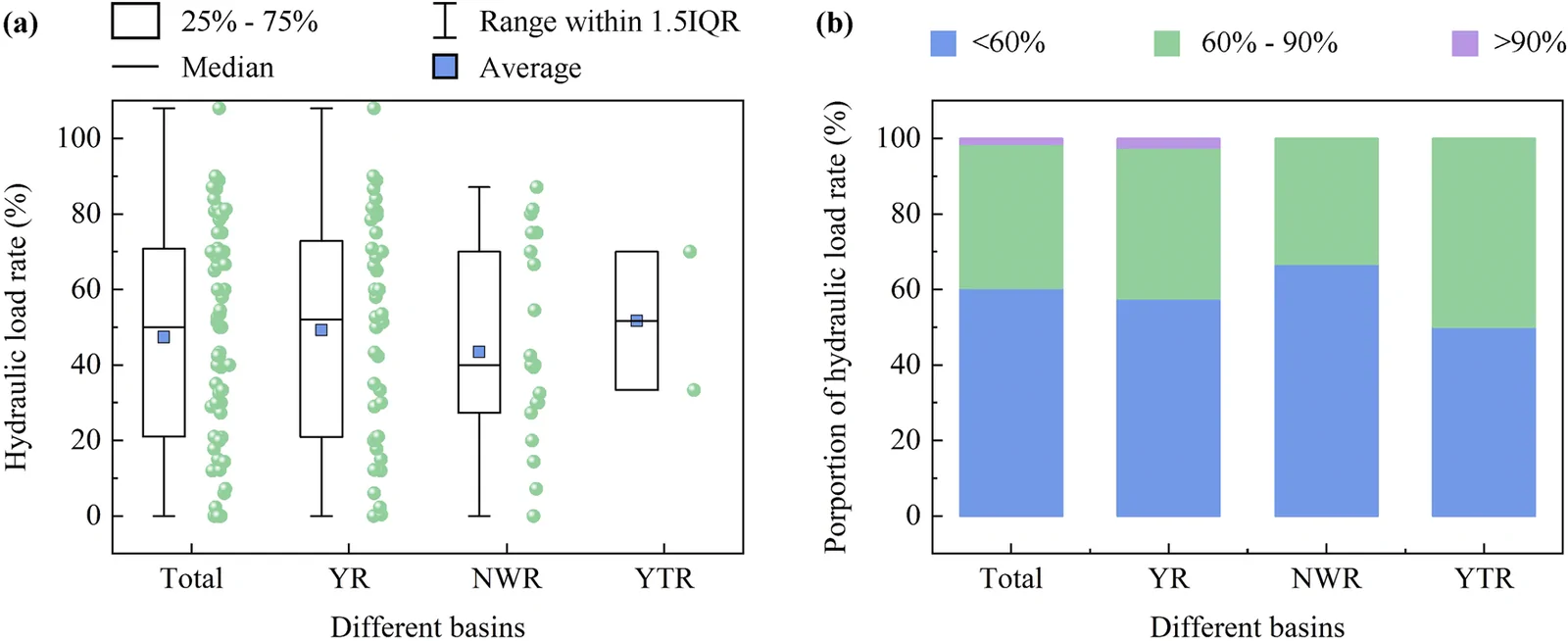
Hydraulic load rate **(a)** and proportion of different hydraulic load rate **(b)** of WWTPs in a multi-river province of China.

In summary, the Gansu WWTPs had a low wastewater collection rate because of the insufficient number of enterprises in industrial parks, which resulted in the widespread occurrence of low-load operations across the province, particularly in the NWR basin, followed by the YR and YTR basins. Fortunately, high-load operational problems were not particularly prominent, except in the YR basin.

#### 3.2.2 Wastewater quality load rate

BOD_5_, COD_Cr_, and NH_3_-N were chosen to investigate the wastewater quality load rate (i.e., water quality problems) of WWTPs at both the provincial and basin scales after investigating the hydraulic load rate. Therefore, low-load and high-load operation states were defined as the actual value (AV) to the design value (DV) of pollutants of <0.6 and >1.2, respectively (Ministry of Housing and Urban-Rural Development, 2015), which can induce excessively low or high growth rate of activated sludge due to the nutrient content of influent pollutants, further result in inadequate pollutant removal efficiency and possible excessive discharge of effluent (Sun et al., 2016). Different from the COD_Cr_ and NH_3_-N, regarding the absence of the BOD_5_ DV in this field document and the national requirements for the WWTPs with an influent BOD_5_ concentration below 100 mg/L (such WWTPs are required to formulate improvement scheme, Xu et al., 2022), a BOD_5_ load rate <1.0 (i.e., the ratio of AV to the value of 100 mg/L) is considered a low-load operation.

The load rates of BOD_5_, COD_Cr_, and NH_3_-N in Gansu WWTPs ranged from 0.3 to 3.9, 0.2 to 2.0, and 0.2 to 1.9, respectively, with average values of 1.7, 0.8, and 0.8, respectively (Figures 7a–c). Among them, the proportion of low-load operation WWTPs based on BOD_5_, COD_Cr_, and NH_3_-N were 18.5%, 41.7%, and 28.8%, respectively, while the proportion of high-load operation WWTPs based on COD_Cr_ and NH_3_-N were 16.7% and 15.3%, respectively (Figures 7d–f). Besides, the operational problems of wastewater quality load rate showed the differences in the three basins. For example, (a) the problems of both low-load and high-load operation of pollutants were most prominent in the YR basin, with the proportions of low-load operation of BOD_5_ (19.0%), COD_Cr_ (42.1%), and NH_3_-N (13.6%) and high-load operational problems of COD_Cr_ (21.1%), and NH_3_-N (11.9%); (b) the above-mentioned problems were particularly prominent in the WWTPs in the NWR basin, with the proportions of low-load operation of BOD_5_ (16.7%), COD_Cr_ (40.0%), and NH_3_-N (11.9%) and high-load operation of COD_Cr_ (10.0%), and NH_3_-N (3.4%); and (c) these problems were not obvious in the YTR basin (Figures 7d–f).

**FIGURE 7:**
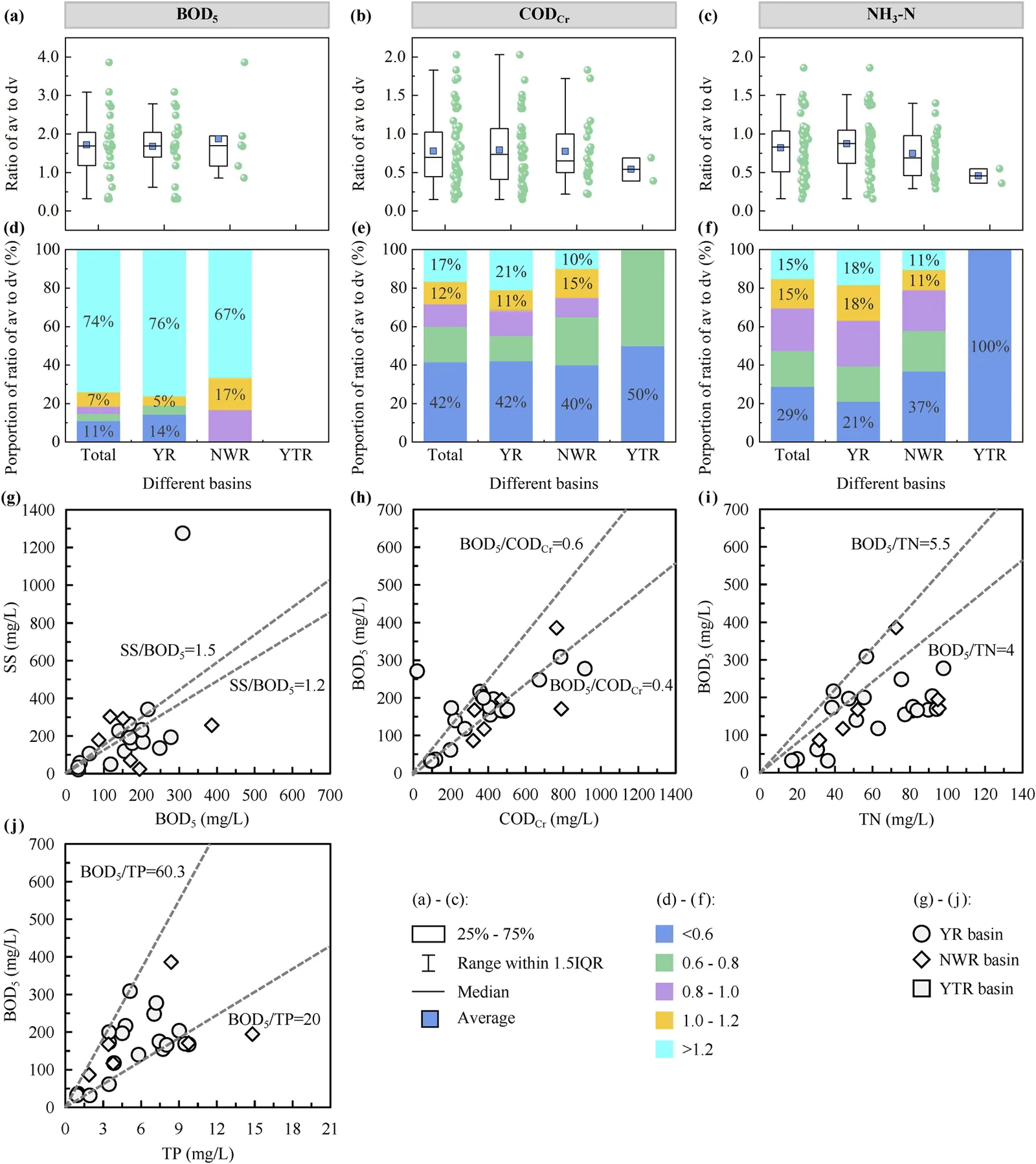
Wastewater quality load rate of main pollutants **(a–c)**, proportion of different wastewater quality load rate of main pollutants **(d–f)**, and ratio between main pollutants **(g–j)** of industrial WWTPs in a multi-river province of China.

The WWTPs in the YR basin primarily exhibited the problems of low-load operation, accompanied by certain high-load operational issues. These problems were more pronounced in the YR basin than those in the NWR and YTR basins.

#### 3.2.3 Wastewater quality characteristics

A balanced relationship among carbon, nitrogen and phosphorus in the influent of WWTPs is crucial for the effective biodegradation processes (Sun et al., 2016). The SS/BOD_5_, BOD_5_/COD_Cr_, BOD_5_/TN, and BOD_5_/TP ratios were selected to study the effects of influent SS on the sludge yield and activity, influent biodegradability, carbon source availability during denitrification, and the feasibility of biological phosphorus removal, respectively. The corresponding reasonable range were 1.2–1.5, 0.4–0.6, >4.0, and >20, respectively (Qi et al., 2020; Sun et al., 2016).

The SS/BOD_5_, BOD_5_/COD_Cr_, BOD_5_/TN, and BOD_5_/TP of Gansu WWTPs were 0.12–4.13, 0.22–13.40, 0.88–5.52, and 13.14–60.30, respectively, with average values of 1.27, 0.90, 2.78, and 31.74 respectively. However, WWTPs with low biological nitrogen removal efficiency (SS/BOD_5_ > 1.5), low influent biodegradability (BOD_5_/COD_Cr_ < 0.4), low carbon source availability during denitrification (BOD_5_/TN < 4), and low substrate availability for polyphosphate-accumulating bacteria during the aerobic phosphorus removal (BOD_5_/TP < 20) accounted for 14.3%, 23.8%, 31.7%, and 9.5%, respectively (Figures 7g–j). Regarding the three problems of low-efficiency biological nitrogen removal, low influent biodegradability, low carbon source availability during denitrification, the proportions of affected WWTPs were higher in the YR basin (15%, 29.3%, and 39.0%, respectively) than in the NWR basin (14.3%, 15.0%, and 25%, respectively). An exception was the problem of low substrate availability for polyphosphate-accumulating bacteria during the aerobic phosphorus removal. In addition, the attributes of WWTPs in the YR basin could not be determined because of the lack of influent BOD_5_ data.

Therefore, based on the survey of wastewater quality characteristics of WWTPs at both the provincial and basin scales, the main operational problems included low-efficiency biological nitrogen removal, low influent biodegradability, and low carbon source availability during denitrification, along with the less prominent issue of low substrate availability for polyphosphate-accumulating bacteria during aerobic phosphorus removal. Further, these three major problems were more pronounced in the YR basin than in the NWR basin.

### 3.3 Response of WWTP operations to major influencing factors

The hydraulic load rate, BOD_5_ pollution load rate, and BOD_5_/COD_Cr_ were the three main drivers for the WWTP operations based on the stepwise forward analysis of the RDA (Supplementary Table S1). Furthermore, the detailed effect of each factor on the operational performance of the WWTPs were explored using Pearson’s correlations (Figure 8). The hydraulic load rate had a significant influence on various aspects of the actual operational performance of the Gansu WWTPs, including pollution control (i.e., removal and compliance rates), unit sludge yield, energy consumption, and operation cost (P < 0.05), implying that it is a key factor affecting the WWTP operational performance. The wastewater quality load rates, including BOD_5_, COD_Cr_, and NH_3_-N, also showed a marked impact on the removal and compliance rates (P < 0.05). Among the wastewater quality characteristics, BOD_5_/COD_Cr_ and BOD_5_/TN were significantly positively correlated with the removal rate; however BOD_5_/TP was negatively correlated with the unit sludge yield (P < 0.05).

**FIGURE 8:**
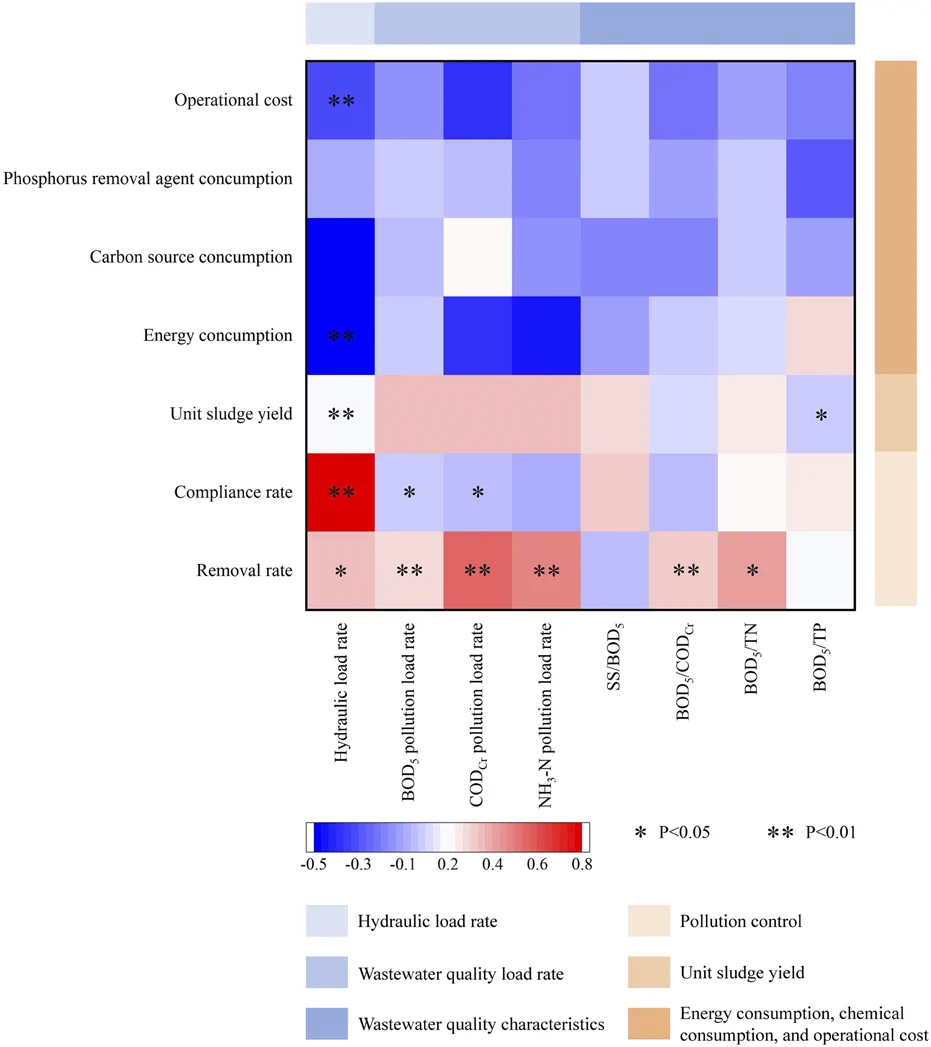
Person’s analyses showing the relations between main operational drivers and actual operational performance of industrial WWTPs in a multi-river province of China.

The above two analyses indicate that the hydraulic load rate, BOD_5_ pollution load rate, and BOD_5_/COD_Cr_ are key factors influencing WWTP operational performance, particularly with respect to low wastewater collection rate and low BOD_5_ pollution load rate. Furthermore, improvements in operation and management practices should be prioritized alongside efforts to address low influent quantity and quality.

## 4 Discussion

### 4.1 Key operational problems of the WWTPs

This section discussed the stepwise operational issues at both provincial and basin scales from design, influent to actual operation of industrial WWTPs, and an overview of operational problems was developed (Figure 2). This section aimed to: (a) identify the special and common problems of WWTP operations in different basins; (b) determine the reasons for low-effectiveness and high-cost operations; and (c) effectively connect the problems with suggestions.

First, the unreasonable design and construction of WWTPs pose a high potential risk of river water pollution under low-level WWTP operations. For example, the industrial WWTPs in Gansu Province showed a dense distribution pattern (0.5–3.0 km of the median distance) and high treatment dependence rate (34.8%–59.9%) across the three basins, especially in the YR and YTR basins. However, municipal WWTPs could not effectively treat due to the lack of associated pretreatment units, except for process upgrading (Xu et al., 2020). Furthermore, a high proportion of medium- and large-scale WWTPs (>1.0 × 10^4^ m^3^/d) was found in the YR basin (48.0%), followed by high proportions of traditional-process-type WWTPs (7.1%) and low-discharge-standard WWTPs (11.9%), respectively. The industrial WWTPs in Gansu Province showed issues related to dense distribution across the basins, delayed construction, unreasonable process design, and low rates of retrofitting and upgrading, particularly in the YR basin.

Second, the underestimated quantity and quality of the influent resulted in a series of low-load operational problems in WWTPs. For instance, the average hydraulic loads of the Gansu WWTPs were only about 47.4%. The low-load operational problems of wastewater quantity were common across all three basins (50.0%–57.5%). Similarly, low-load operational problems of wastewater quality remained dominant (BOD_5_:18.5%, COD_Cr_: 41.7%, and NH_3_-N: 28.8%) in all three basins. Moreover, three problems involving wastewater quality characteristics were noteworthy: low-efficiency biological nitrogen removal (14.3%), low influent biodegradability (23.8%), and low carbon source availability during denitrification (31.7%), particularly in the YR basin. Therefore, to further enhance the treatment efficiency of WWTPs, the Chinese government required WWTPs with influent BOD_5_ concentrations below 100 mg/L to formulate improvement schemes (Xu et al., 2022); however, the proportion of such WWTPs remained high across the different basins (16.7%–19.0%), particularly in the YR basin. As a result, the pollution control performance of the WWTPs in the YR basin was poorer than that of the other two basins. Among the studied indices, TN and TP showed the lowest removal rates (<90%), particularly in the YR basin. A low compliance-rate was also observed in the YR (35.9%) and NWR (33.0%) basins. The main reasons for this were as follows: (1) the unreasonable design, such as the high treatment dependence rate and low process upgrading rate of WWTPs in the YR basin, owing to the limited treatment efficiency of traditional processes for difficult-to-biodegrade wastewater (Abreham Awono and Adam, 2025; Hu et al., 2018); and (2) the low-load problems in terms of influent quantity and quality of WWTPs in the YR basin, which is in agreement with our study results and other reports (Hu et al., 2018). In brief, problems of influent quantity and quality were common across all three basins, particularly limited the nitrogen and phosphorus removal efficiency of WWTPs. These issues were more pronounced in the YR basin.

Finally, low-level operation led to a series of actual operational problems in WWTPs, apart from the above-mentioned pollution control performance. In detail, the four operational parameters of WWTPs (i.e., unit sludge yield, energy consumption, chemical consumption, and operational cost) exhibited the poorest performance in the NWR basin, and the possible reasons included: (1) the widespread application of high-unit-sludge-yield techniques (e.g., OD and AAO) and high-complexity combined processes (e.g., Membrane Bio-Reactor and Sedimentation Tank+Advanced Oxidation Process+Filters); (2) the low-load problems of influent quantity and quality, such as WWTPs with 66.7% of low-hydraulic-load operation and 40.0% of low-load operation of COD_Cr_; (3) the low-level operation, such as the widespread addition of agents (e.g., 79.2% of carbon source addition and 66.7% of phosphorus removal agent addition); and (4) another important factor from climatic impacts, such as the increases in energy consumption and relevant operational cost induced by WWTP insulation due to low air temperature and its long duration period, agreed by the result that the energy consumption in the upstream provinces of the YRB is higher than these in their middle and downstream provinces (Xu et al., 2022). In conclusion, the operational performance problems of industrial WWTPs were evident at both the provincial and basin scales. However, the main operational issues differed among the basins, with WWTPs in the YR basin exhibiting low pollution control performance and those in the NWR basin showing high unit sludge yield, energy consumption, chemical consumption, and operational costs.

### 4.2 Management recommendations for the WWTP operations

Based on the above analysis of the operation issues, following one-to-one management recommendations are proposed (Figure 2).

First, process design should be improved and upgraded, particularly in the YR basin. Furthermore, three major problems require priority attention: high treatment dependence rate, low process upgrading rate, and low discharge standard of WWTPs, in addition to other challenges that may be more difficult to address in the short term. Therefore, the following detailed recommendations are proposed to address these issues: (1) Increasing the pretreatment units for industrial wastewater treatment. Currently, most industrial wastewater treatments rely on municipal WWTPs; however, most operate at a low hydraulic load. Therefore, the construction of industrial WWTPs should be carefully planned and implemented. The pretreatment units should be built before municipal WWTPs when operational loads are less than 90%. Otherwise, it is recommended that industrial WWTPs be constructed, considering the insufficient treatment and operational abilities of municipal WWTPs (Liu et al., 2025). (2) Optimizing the pre- and post-treatment units to simplify and enhance the effectiveness of the design processes. During pretreatment, it has been suggested that converting high-efficiency filtering or settling processes (e.g., membrane grille or high-efficiency settling tanks) into high-efficiency anaerobic tank processes increases the availability of carbon sources for secondary treatment units (Liu et al., 2025; Wei et al., 2022). During advanced treatment, we suggest replacing the high-energy and high-complexity processes with a combination of low-energy and simplified process using artificial wetlands (Küçükbayrak and Alver, 2025; Mei et al., 2025), particularly for water-reuse WWTPs. Using these two methods, high chemical and energy consumption can be avoided, promoting a low-carbon design and operation. (3) Accelerating the upgrading of traditional technologies. At present, the proportion of high-water-pollution-risk WWTPs (i.e., outdated discharge standards, outdated technology, and direct discharge of pollutants into rivers) remains relatively high, mainly in the YR basin. Therefore, upgrading and renovation of such WWTPs should be accelerated to ensure compliance with the Class 1A standard of GB 18918-2002.

Second, the low-load operational problems of influent quantity and quality should be prioritized, owing to the inadequate number of enterprises into industrial parks and the unreasonable industry layout, which further limits the actual operational performance, especially in the YR basin (Supplementary Table S1; Figure 2). To this end, efforts should be made in the following three aspects. (1) Enhancing the collection rate of wastewater and number of enterprises in industrial parks to address the problems of low hydraulic loads. First, a detailed investigation of drainage enterprises, furtive drain outlets, and pipelines should be conducted to guarantee all wastewater in WWTPs and the healthy operation of pipelines, especially for the YR and NWR basins. However, efforts to attract local and out-of-town enterprises into parks must be strengthened. (2) Optimizing the current industrial layout of parks to address the problems of low wastewater quality load rate and low concentration of influent BOD_5_. Some industries that discharge highly biodegradable wastewater, such as food processing and agricultural by-product processing, need to be introduced in the three basins to improve wastewater quality (Wei et al., 2021). (3) Improving the operational levels to increase the overall compliance rate of WWTPs and reduce the number of unstable compliance indices. Compliance-rate problems involving WWTPs and their key indicators are not only related to low operational loads but also to low operational levels. However, the low-load operation problems of influents cannot be effectively resolved in the short term; therefore, professional operation routes should be emphatically built by introducing professional operators and conducting professional operation training, particularly in the YR basin.

Finally, the operation and management models of WWTPs should be systematically optimized to effectively reduce operational costs. The current management mechanism of Gansu WWTPs is mainly based on supervision, focusing on wastewater collection and final discharge compliance while neglecting the improvements in operational effectiveness (Revollar et al., 2020). To meet the increasingly stringent discharge standards, following measures have been implemented: (a) the widespread application of high-complexity combined processes; (b) increasing chemical consumption; (c) the above two results further led to the increases in energy consumption, unit sludge yield, and overall operational costs (Rezzoug et al., 2025), especially in the NWR basin. Hence, three specific suggestions are proposed to promote a transition from effluent compliance to full-process optimization. (1) Enhancing the upgrading of high-sludge yield techniques and reducing the unit sludge yield. The high sludge yields of the Gansu WWTPs were mainly due to the popular use of high-sludge-yield techniques, high-complexity combined processes, and agents. Therefore, some changes are required from both short-term and long-term perspectives. First, the operating conditions of the processes should be optimized to reduce the amount of the chemical consumption, particularly the phosphorus removal agent. Second, low-carbon and low-sludge-yield techniques should be gradually introduced, while industries producing highly biodegradable wastewater should be encouraged to enter the industrial parks to reduce the need for carbon source addition and associated increase in the sludge yield. (2) Strengthening reductions in energy consumption, material consumption, and operational costs. Currently, the costs of energy consumption, material consumption, and sludge disposal account for approximately 47.8% of total operational costs. In the short term, efforts should be made to promote the upgrade of facilities with the advantages of energy savings, high-efficiency sludge disposal, and the rational addition of agents to optimize operational costs. In the long term, efforts should be intensified to gradually introduce high-quality carbon source industries and upgrade high-energy consumption processes to maximize the reduction in operational costs. (3) Transforming the operation and management model from effluent compliance to full-process optimization. After a decade of efforts, industrial wastewater treatment has advanced from the construction of WWTPs to the high-quality operation. However, WWTP management primarily focuses on wastewater collection and final discharge compliance, with relatively limited attention paid to the overall operational effectiveness. Hence, low-carbon operational policies should be introduced to support a more balanced management approach that integrates wastewater collection, discharge compliance, and operational efficiency.

## 5 Conclusion

Based on a long-term field investigation of industrial WWTPs in a multi-river province with three main basins, four key conclusions were drawn.

1. An overview of operational problems involving three major and 15 specific aspects was developed at both the basin and provincial scales. Therein, the most prominent problems included high treatment dependence rate (50.0%), high proportion of WWTPs operating under low influent quantity (47.4%) and quality loads (BOD_5_:18.5%, COD_Cr_: 41.7%, and NH_3_-N 28.8%), a high proportion of WWTPs with a compliance rate <90% (34.4%), and high unit sludge yield (2.02 t/10^4^ m^3^), and high operation costs (3.91 CNY/m^3^).
2. Differences in the prominent operational problems of the Gansu WWTPs were observed across the three basins. The major ones in the YR basin involved design- and influent-related issues, including a high treatment dependence rate. However, the operational problems of WWTPs involving influent-related and actual operational performance were predominant in the NWR basin. Compared with the above two basins, the operational problems in the NWR basin were relatively less prominent.
3. Low influent quantity and quality loads were the primary factors influencing the operational performance of the Gansu WWTPs, followed by the operational level and design. Based on these findings, specific recommendation are proposed, including optimizing operational levels, increasing influent loads in terms of quantity and quality, and addressing design deficiencies to facilitate the transition from an effluent compliance-oriented approach to comprehensive process-based management.
4. This study provides a useful framework for investigating the industrial WWTPs, particularly for identifying common and specific operational issues, thereby supporting the development of targeted countermeasures to reduce river water environmental risks associated with WWTP discharge.

## Data Availability

The original contributions presented in the study are included in the article/Supplementary Material, further inquiries can be directed to the corresponding authors.
