# Supplementary material for: Operational challenges of industrial wastewater treatment plants in a multi-river province of China: implications for problem identification and management
Source: Front Chem. 2026 Sep 23;14:1955505. doi: 10.3389/fchem.2026.1955505 (PMC13642841; doi:10.3389/fchem.2026.1955505)
Supplement: Supplementary file 1 [file DataSheet1.docx]

**Supplementary Materials**

**Operational challenges of industrial wastewater treatment plants in a multi-river Province of China: Implications for problem identification and management**

Zhilong Dong^1*^, Jing Zhou^2,3^, Nan Li^4,5,^*, Yi Huang^2,3^, Fengli Tang^2,3^, Long Sun^2,3^, Wei Tao^2,3^, Bowen Dong^2,3^, Chengcheng Wen^2,3^

^1^Gansu Appraisal Center for Ecology Environment & Engineering, Lanzhou, China

^2^Gansu Academy of Eco-environmental Sciences, Lanzhou, PR China

^3^Key Laboratory of Industrial Waste Recycling and Regulation, Gansu Academy of Eco-environmental Sciences, Lanzhou, PR China

^4^Key Laboratory of Northwest Water Resource, Environment and Ecology, MOE, Xi'an University of Architecture and Technology, Xi’an, PR China

^5^Shaanxi Provincial Field Scientific Observation and Research Station of Water Quality in Qinling Mountains, Xi’an University of Architecture and Technology, Xi’an, PR China

***Correspondence:**

Zhilong Dong, [hitzhilongdong@163.com](mailto:hitzhilongdong@163.com)

Nan Li, [linan@xauat.edu.cn](mailto:huangtinglin@xauat.edu.cn)

**The number of pages:** total 8 pages including 1 pages of 1 SI Table and 5 pages of 5 SI Figures.

**Fig.S1** Published papers on the theme of wastewater treatment plants’ (WWTPs) operaion in China during 2005 to 2025.

**Fig.S2** Construction (a) and distribution (b) of industrial WWTPs and the distance between WWTPs and rivers (c) in a multi-river province of China.

**Fig.S3** Design capacity (a) and its proportion (b) of industrial WWTPs in a multi-river province of China. Here, based on the design capacity, the industrial WWTPs can be divided into the four levels including micro scale (<0.5×10^4^ m^3^/d), small scale [(0.5~1)×10^4^ m^3^/d], medium scale [(1~10)×10^4^ m^3^/d], and large scale (>10×10^4^ m^3^/d).

**Fig.S4**  Type and proportion of main treatment processes of industrial WWTPs in a multi-river province of China. **Note:** Sedimentation Tank (ST); Advanced Oxidation Process (AOPs); Anoxic-Oxic Process (AO); Anaerobic-Anoxic-Oxic Process (AAO); Membrane Bio-Reactor (MBR); Oxidation Ditch (OD); Sequencing Batch Reactor Activated Sludge Proces (SBR).

**Fig.S5** Proportion of discharge standard of pollutants for industrial WWTPs in a multi-river province of China. **Note:** Discharge standard of pollutants for municipal wastewater treatment plant (DSPMWTP); Environmental quality standards for surface water (EQSSW); Integrated wastewater discharge standard (IWDS); Emission standard of pollutants for petroleum chemistry industry (ESPPCI); Discharge standard of Water pollutants for fermentation alcohol and distilled spirits industry (DSWPFADSI).

**Table S1** Key influencing factors explaining the WWTPs’ operation using stepwise forward analysis of Redundancy analysis (RDA) in a multi-river province of China.

| Explanation for the WWTP operation | Factors | Explanation | Contribution | *P* |
| --- | --- | --- | --- | --- |
| 17.6% | Hydraulic load rate | 8.3% | 47.3% | 0.03 |
|  | BOD_5_ pollution load rate | 3.2% | 18.5% | 0.20 |
|  | BOD_5_/COD_Cr_ | 1.6% | 9.1% | 0.39 |
|  | BOD_5_/TP | 1.5% | 8.5% | 0.40 |
|  | BOD_5_/TN | 1.5% | 8.5% | 0.41 |
|  | NH_3_-N pollution load rate | 0.7% | 3.8% | 0.67 |
|  | COD_Cr_ pollution load rate | 0.5% | 2.7% | 0.72 |
|  | SS/BOD_5_ | 0.3% | 0.1% | 0.87 |
